# *Escherichia coli* O104 Associated with Human Diarrhea, South Africa, 2004–2011

**DOI:** 10.3201/eid1808.111616

**Published:** 2012-08

**Authors:** Nomsa P. Tau, Parastu Meidany, Anthony M. Smith, Arvinda Sooka, Karen H. Keddy, for the Group for Enteric, Respiratory, and Meningeal Disease Surveillance in South Africa

**Affiliations:** National Health Laboratory Services, Johannesburg, South Africa (N.P. Tau, A.M. Smith, A. Sooka, K.H. Keddy);; University of the Witwatersrand, Johannesburg (P. Meidany, A.M. Smith, K.H. Keddy)

**Keywords:** Escherichia coli, diarrheagenic E. coli, E. coli O104, South Africa, outbreak, human diarrhea, bacteria

## Abstract

To determine the origin of >4,000 suspected diarrheagenic *Escherichia coli* strains isolated during 2004–2011 in South Africa, we identified 7 isolates as serotype O104; 5 as enteroaggregative *E. coli* O104:H4, and 2 as enteropathogenic *E. coli* O104:non-H4. Pulsed-field gel electrophoresis showed that these isolates were unrelated to the 2011 *E.* coli O104:H4 outbreak strain from Germany.

*Escherichia coli* is the predominant microorganism of human colonic flora (*1*). It is mostly harmless to the intestinal lumen. However, some strains (diarrheagenic *E. coli* [DEC]) can cause disease ranging from moderate-to-severe diarrhea with complications to hemolytic uremic syndrome (*1*).

During May–June 2011, an outbreak of bloody diarrhea and hemolytic uremic syndrome occurred in Germany and other parts of Europe (*2*). The Shiga toxin–producing *E. coli* (STEC) serotype O104 strain was the etiologic agent of this outbreak and accounted for >4,000 cases and 50 deaths (*3*). This outbreak strain showed an unusual combination of virulence factors of STEC and enteroaggregative *E. coli* (EAggEC). Furthermore, the outbreak strain showed extended spectrum β-lactamase (ESBL) activity (*2**,**4*).

Before the 2011 outbreak, *E. coli* O104 had been reported in parts of Europe and South Korea (*5**,**6*). In South Africa, data for *E. coli* serotypes are scarce. However, because questions arose about the ancestral origin of the 2011 outbreak strain from Germany, we investigated the occurrence of *E. coli* O104 associated with human diarrhea in 2 surveillance programs for enteric pathogens in South Africa during 2004–2011. We also investigated phenotypic and genotypic properties of *E. coli* O104 strains from South Africa and compare these with properties of the outbreak strain from Germany.

## The Study

The Centre for Enteric Diseases (CED) of the National Institute for Communicable Diseases in South Africa is a reference center for human infections involving enteric pathogens including DEC, *Salmonella* spp., *Shigella* spp., and *Vibrio cholerae*, and it participates in national laboratory-based surveillance for these pathogens. Isolates are voluntarily submitted to the CED from ≈200 clinical microbiology laboratories across the country. The CED also recovers enteric pathogens through its involvement in the Rotavirus Surveillance Project, which started in mid-2009 and involves 5 sentinel hospital sites in South Africa. This project is involved with identification of enteric pathogens (bacterial, viral, and parasitic) associated with diarrhea in children <5 years of age.

All suspected DEC isolates received at the CED were identified by using standard microbiological identification techniques. Serotyping of O antigen was performed by using tube agglutination as described (*7*). Presence of H4 antigen was determined by PCR detection of the *fliC*_H4_ gene (*8*). Resistance to antimicrobial drugs (ampicillin, amoxicillin/clavulanic acid, sulfamethoxazole/trimethoprim, chloramphenicol, nalidixic acid, ciprofloxacin, tetracycline, kanamycin, imipenem, ceftriaxone, and ceftazidime) was determined by using Etests (bioMérieux, Marcy l’Etoile, France), and ESBL activity was investigated by using double-disk synergy methods, as described by Clinical and Laboratory Standards Institute (Wayne, PA, USA) 2009 guidelines (*9*).

PCR was used to distinguish DEC strains from nonpathogenic *E. coli* strains. The PCR consisted of 3 multiplex reactions with published primer sequences. Reactions included 0.2 μmol/L of each primer (Table 1) and 1.5 mmol/L MgCl_2_. PCR thermal cycling included 35 cycles at 94°C for 1.5 min, 60°C for 1.5 min, and 72°C for 1.5 min.

**Table 1:** PCR primers used for amplification of *Escherichia coli* genes, South Africa

Gene target*	Primer sequence, 5′ → 3′	Size of amplification product, bp	Multiplex PCR in which primers are included	Reference
*stx1*	CAGTTAATGTGGTGGCGAAGG	348	A	(*10*)
	CACCAGACAATGTAACCGCTG		A	(*10*)
*stx2*	ATCCTATTCCCGGGAGTTTACG	584	A	(*10*)
	GCGTCATCGTATACACAGGAGC		A	(*10*)
*eae*	TCAATGCAGTTCCGTTATCAGTT	482	A	(*11*)
	GTAAAGTCCGTTACCCCAACCTG		A	(*11*)
*est*	ATTTTTCTTTCTGTATTGTCTT	190	B	(*12*)
	CACCCGGTACAAGCAGGATT		B	(*12*)
*elt*	GGCGACAGATTATACCGTGC	440	B	(*12*)
	CGGTCTCTATATTCCCTGTT		B	(*12*)
*ipaH*	CTCGGCACGTTTTAATAGTCTGG	933	C	(*11*)
	GTGGAGAGCTGAAGTTTCTCTGC		C	(*11*)
*aat*	CTGGCGAAAGACTGTATCAT	630	C	(*13*)
	CAATGTATAGAAATCCGCTGTT		C	(*13*)
*daaC*	CAGGTCATCCGGTCAGTCGG	212	C	This study
	CAATGCCACGTACAACCGGC		C	This study

*stx, Shiga toxin; *eae*, intimin outer membrane protein; *est*, heat-stable enterotoxin; *elt*, heat-labile enterotoxin; *ipaH*, invasion protein; *aat*, transporter protein; *daaC*, accessory protein with a function in F1845 fimbriae production.

An isolate was identified as STEC if a PCR result was positive for Shiga toxin genes 1 or 2 (*stx1* or *stx2*); as enterohemorrhagic *E. coli* if a PCR result was positive for the gene coding intimin outer membrane protein and an *stx* gene; as enteropathogenic *E. coli* (EPEC) if a PCR result was positive for the gene coding intimin outer membrane protein; as enterotoxigenic *E. coli* if a PCR result was a positive for genes coding heat-stable enterotoxin or heat-labile enterotoxin; as enteroinvasive *E. coli* if a PCR result was positive for the gene coding an invasion protein; as EAggEC if a PCR result was positive for the gene coding a transporter protein; and as diffusely adherent *E. coli* if a PCR result was positive for the gene coding an accessory protein with a function in F1845 fimbriae production. These genes are listed in Table 1.

Genotypic relatedness of strains was investigated by using a PulseNet protocol (*14*) for pulsed-field gel electrophoresis (PFGE) of *Xba*I-digested genomic DNA in a CHEF-DR III electrophoresis system (Bio-Rad Laboratories, Hercules, CA, USA) and the following electrophoresis parameters: voltage 6 V, run temperature 14°C, run time 19 h, initial switch time 6.76 s, final switch time 35.38 s, and included angle 120°. PFGE patterns were analyzed by using BioNumerics version 6.5 software (Applied Maths, Sint-Martens-Latem, Belgium). Dendrograms of patterns were created by using unweighted pair group method with arithmetic averages. Analysis of band patterns incorporated the Dice coefficient at an optimization setting of 1.5% and a position tolerance setting of 1.5%.

During January 2004–May 2011, CED received >4,000 suspected DEC isolates for further laboratory characterization. Of these isolates, 7 (0.2%) were serotype O104. These isolates were collected from Gauteng (n = 3), Mpumalanga (n = 2), and Eastern Cape (n = 1) and North West (n = 1) Provinces of South Africa. *E. coli* O104 was isolated more often from children <2 years of age than from older children and adults (Table 2). There were more female patients (57%) affected than male patients (43%). Five (71%) of 7 isolates were EAggEC, and 2 (29%) of 7 isolates were EPEC (Table 2). PCR amplification of the *fliC_H4_* gene showed that all EAggEC isolates produced H4 antigen and that all the EPEC isolates did not produce H4 antigen. Therefore, all EAggEC were serotype O104:H4 and all EPEC were serotype O104:non-H4.

**Table 2:** *Escherichia coli* O104 strains associated with human diarrhea, South Africa, 2004–2011*

Strain no.	Collection date	Province	Patient age/sex	*E. coli* pathotype	H antigen	Antimicrobial drug susceptibility†
1	2007 Feb 23	Eastern Cape	4 mo/F	EAggEC	H4	R^Amp^ S^Amc^ R^Sxt^ S^Chl^ S^Nal^ S^Cip^ R^Tet^ S^Kan^ S^Str^ S^Imi^ S^Cro^ S^Caz^
2	2007 Nov 16	Mpumalanga	43 y/M	EAggEC	H4	R^Amp^ S^Amc^ R^Sxt^ S^Chl^ S^Nal^ S^Cip^ R^Tet^ S^Kan^ R^Str^ S^Imi^ S^Cro^ S^Caz^
3	2009 Apr 12	Gauteng	10 mo/F	EPEC	Non-H4	S^Amp^ S^Amc^ S^Sxt^ S^Chl^ S^Nal^ S^Cip^ S^Tet^ S^Kan^ S^Str^ S^Imi^ S^Cro^ S^Caz^
4	2010 Feb 24	Mpumalanga	7 mo/M	EAggEC	H4	R^Amp^ S^Amc^ R^Sxt^ S^Chl^ S^Nal^ S^Cip^ S^Tet^ S^Kan^ S^Str^ S^Imi^ S^Cro^ S^Caz^
5	2010 Mar 3	North West	7 mo/F	EAggEC	H4	R^Amp^ S^Amc^ R^Sxt^ S^Chl^ S^Nal^ S^Cip^ R^Tet^ S^Kan^ S^Str^ S^Imi^ S^Cro^ S^Caz^
6	2010 Jun 16	Gauteng	12 mo/M	EAggEC	H4	R^Amp^ S^Amc^ R^Sxt^ S^Chl^ S^Nal^ S^Cip^ S^Tet^ S^Kan^ R^Str^ S^Imi^ S^Cro^ S^Caz^
7	2010 Jul 17	Gauteng	15 mo/F	EPEC	Non-H4	S^Amp^ S^Amc^ S^Sxt^ S^Chl^ S^Nal^ S^Cip^ S^Tet^ S^Kan^ S^Str^ S^Imi^ S^Cro^ S^Caz^

*EaggEC, enteroaggregative *E. coli*; EPEC, enteropathogenic *E. coli*. †R, resistant; S, susceptible. Isolates were classified as antimicrobial drug resistant at the following MIC breakpoint concentrations: ampicillin (Amp), MIC >16 μg/mL; amoxicillin/clavulanic acid (Amc), MIC >16 μg/mL; sulfamethoxazole/trimethoprim (Sxt), MIC >4 μg/mL; chloramphenicol (Chl), MIC >16 μg/mL; nalidixic acid (Nal), MIC >32 μg/mL; ciprofloxacin (Cip), MIC >2 μg/mL; tetracycline (Tet), MIC >8 μg/mL; kanamycin (Kan), MIC >32 μg/mL; streptomycin (Str), MIC >64 μg/mL; imipenem (Imi), MIC >8 μg/ml; ceftriaxone (Cro), MIC >16 μg/mL; ceftazidime (Caz), MIC >16 μg/mL.

EPEC isolates were susceptible to all antimicrobial drugs tested (Table 2). EAggEC isolates were susceptible to amoxicillin/clavulanic acid, chloramphenicol, nalidixic acid, ciprofloxacin, kanamycin, imipenem, ceftriaxone and ceftazidime; were resistant to ampicillin and sulfamethoxazole/trimethoprim; and were variably susceptible to tetracycline and streptomycin (Table 2). None of the EPEC or EAggEC isolates showed ESBL activity.

Dendrogram analysis of PFGE patterns showed that EPEC isolates were diverse and clustered at a pattern similarity of 76%, and that EAggEC isolates were highly clonal and clustered at a pattern similarity of 90% (Figure). The PFGE pattern of the 2011 outbreak strain from Germany did not match those of strains from South Africa. Therefore, the outbreak strain from Germany was determined to be unrelated to strains from South Africa. However, the strain from Germany was most closely related to the EAggEC strain cluster from South Africa (pattern similarity 85%).

**Figure:**
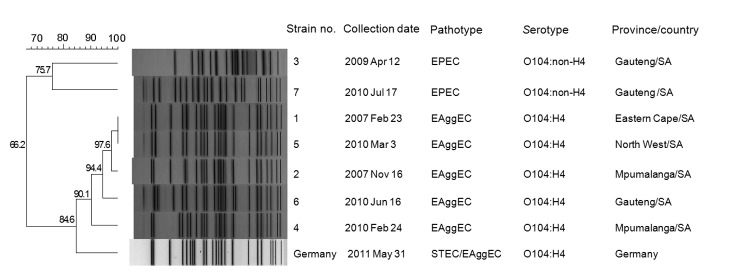
Pulsed-field gel electrophoresis profiles (*Xba*I digestion) of *Escherichia coli* O104 strains from South Africa (SA) compared with a strain from Germany. EPEC, enteropathogenic *E. coli*; EaggEC, enteroaggregative *E. coli*; STEC, Shiga toxin–producing *E. coli*. Scale bar and numbers along branches indicate percentage pattern similarity.

## Conclusions

Our findings show that *E. coli* O104 is rarely associated with human diarrhea in South Africa and accounts for <1% of all DEC pathotypes identified during 2004–2011 by laboratory-based surveillance and limited sentinel surveillance. Strains of *E. coli* O104 from South Africa were mostly associated with EAggEC pathotypes; most (5/7) were identified as EAggEC. Infection with this pathotype has been associated with more persistent diarrhea (duration >14 days) (*15*). Therefore, patients infected with EAggEC are more likely to have fecal cultures tested, potentially leading to greater numbers of EAggEC isolates identified.

In South Africa, *E. coli* O104 infections were more commonly identified in children than in adults. Unlike the *E. coli* O104 strain that caused the outbreak in Germany, strains of *E. coli* O104 from South Africa did not produce Shiga toxin and did not show ESBL activity. PFGE data supported these phenotypic data, suggesting that strains from South Africa were not related to the outbreak strain from Germany. The PFGE data also showed that strains of EAggEC O104:H4 from South Africa were highly clonal. Further work is necessary to better understand the global distribution of these isolates and the role of molecular epidemiologic techniques in characterizing this newly emerging serotype.
